# Long‐Term Treadmill Exercise and Voluntary Running Pre‐Training Attenuates Vascular Dementia‐Related Pathology by Regulating Hippocampal Structural Synaptic Plasticity in a Rat Model

**DOI:** 10.1002/brb3.70833

**Published:** 2025-09-10

**Authors:** Yujiao Li, Yuxuan Li, Linlin Zhang, Kunxia Su

**Affiliations:** ^1^ Public Sports Education Center Zhongyuan Institute of Science and Technology Zhengzhou China; ^2^ School of Physical Education and Health Henan University of Chinese Medicine Zhengzhou China; ^3^ Department of Physical Education Henan Normal University Xinxiang China

**Keywords:** hippocampus, structural synaptic plasticity, treadmill exercise, vascular dementia, voluntary running

## Abstract

**Background:**

Clinical and basic research suggests that exercise is a safe behavioral intervention and effective in improving cognition in vascular dementia (VD). However, despite global efforts, there is still no effective method to completely cure VD. This study aimed to investigate the effects of long‐term exercise pretreatment on typical VD pathology in a rat model, and further compare the neuroprotective impacts of different exercise modalities on VD rats.

**Methods:**

Forty‐eight SD adult male rats (200–240 g) were randomly allocated to a control group (Con, n = 12), a vascular dementia group (VD, n = 12), and a treadmill exercise and vascular dementia group (Tre‐VD, n = 12), a voluntary running and vascular dementia group (Vol‐VD, n = 12). Aerobic exercise training included treadmill exercise in the Tre‐VD rats, and voluntary running in the Vol‐VD rats, respectively. The bilateral common carotid artery occlusion operation was performed to induce the VD rat model in all groups except the Con group. Rat behavior, anxious‐depressive‐like behavior and cognition, was tested by the sucrose preference test, the open field test, the novel object recognition test, and the Y‐maze and passive avoidance test. Brain microdialysis combined with a high‐performance liquid chromatography (HPLC) system was applied to measure neurotransmitter concentrations in hippocampal extracellular fluid, including epinephrine (E), norepinephrine (NE), and dopamine (DA). Immunofluorescent staining was conducted to measure neuronal apoptosis. Transmission electron microscope was carried out to analyze hippocampal synaptic ultrastructure.

**Results:**

The behavioral results also showed that long‐term treatment and voluntary pretreatment significantly alleviated cognitive impairment and anxious‐depressive‐like behaviors in VD rats (*p* < 0.05). Meanwhile, compared to the Con rat, VD rats had significantly decreased E NE, DA, and 5‐HT (*p* < 0.05), while exercise significantly increased those neurotransmitter levels in the hippocampus (*p* < 0.05). Moreover, neuronal apoptosis was found in the VD rat when comparing with the Con group (*p* < 0.05), conversely, exercise pretreatment effectively alleviated VD‐induced neuronal apoptosis (*p* < 0.05). In addition, the results of TEM revealed a significant reduction in hippocampal synapse numbers and postsynaptic density as well as the width of the synaptic cleft in VD rats compared to the Con (*p* < 0.05), which was reversed by eight weeks of exercise pretreatment (*p* < 0.05). Intriguingly, overall speaking, the neuroprotective hippocampal structural synaptic plasticity effect of voluntary running on VD rats is better than treadmill exercise.

**Conclusion:**

Both treadmill exercise and voluntary running improve cognitive function and ameliorate anxious‐depressive‐like behaviors in VD rat models. These therapeutic effects are mediated through enhanced synaptic structural plasticity in the hippocampal region of VD rats.

## Graphical abstract

Bilateral common carotid artery occlusion was used to establish a vascular dementia rat model. The VD rat exhibits impairment of cognitive function and anxious‐depressive‐like behaviors, which is associated with the impairment of hippocampal structural synaptic plasticity. However, both treadmill exercise and voluntary running improve cognitive function and ameliorate anxious‐depressive‐like behaviors in VD rat models, these therapeutic effects are mediated through enhanced synaptic structural plasticity in the hippocampal region of VD rats. Intriguingly, the neuroprotective hippocampal structural synaptic plasticity effect of voluntary running on VD rats is better than treadmill exercise.

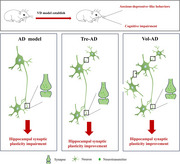

## 1 Introduction

Vascular dementia (VD) is a progressive neurological disease, characterized by irreversible cognitive decline and compromised quality of life (O'Brien and Thomas 2015). Neuronal loss and dysfunction are responsible for the cognitive impairment and behavioral disturbances observed in VD (Kalaria 2018). Diffusion tensor imaging (DTI) analyses reveal significant white matter network deterioration in VD patients, manifested through reduced global efficiency, diminished local efficiency, and lower average clustering coefficients compared to other dementia subtypes, including mild cognitive impairment (MCI), Alzheimer's disease (AD), and mixed dementia (Zhang et al. 2023). Alarmingly, VD affects 1%–4% of individuals aged ≥65 years, and the prevalence appears to double every 5–10 years after the age of 65 years (McVeigh and Passmore 2006). Therefore, these findings underscore the imperative to develop effective strategies for VD prevention and disease‐modifying interventions.

Current pharmacological interventions demonstrate limited clinical generalizability with significant risk of adverse effects, maintaining VD as an intractable therapeutic target despite extensive research spanning decades (Linh et al. 2022, Birks and Flicker 2007). Such limitations highlight the necessity of redirecting research focus toward early‐stage preventive interventions as a pragmatic alternative. Promisingly, accumulating evidence underscores the cost‐effectiveness and prophylactic potential of exercise interventions in VD risk mitigation, showing capacity to delay disease onset and slow progression rates, with exercise‐induced cognitive benefits being robustly validated across animal (Biose et al. 2024) and human models (Chen et al. 2022, Calabrò et al. 2015). Furthermore, mechanistic studies utilizing established VD models—including bilateral common carotid artery occlusion (2‐VO) (Gao et al. 2023, Zhang et al. 2024), four‐vessel occlusion (4‐VO) (Wan et al. 2015, Zhao et al. 2017), and middle cerebral artery occlusion (MCAO) (Gao et al. 2018, Mankhong et al. 2020)—consistently demonstrate exercise‐mediated neuroprotection through multiple pathways. The 2‐VO model remains predominant in VD research due to its technical reproducibility (85% success rate) and precise ischemic control (Zhao et al. 2021). Accordingly, our experimental design employs the 2‐VO method to induce a VD rat model.

The improving effect of exercise intervention on cognitive function has been acknowledged in both animal models and humans. Abundant research has revealed that exercise programs improve balance ability and the activities of daily living in patients of VD or Alzheimer's disease (Yi et al. 2024, Liu‐Ambrose et al. 2016, Aarsland et al. 2010). Data from different VD animal models suggest that exercise‐mediated neuroprotection effect through regulating hippocampal neurogenesis and BDNF expression (Choi et al. 2016) as well as neuronal apoptosis (Gao et al. 2023)—all critical mechanisms underlying VD‐related cognitive deficits. Despite these advances, the cellular and molecular basis for how exercise enhances cognition remains unclear in contemporary research. The maintenance of stable synaptic structure, known to be essential for memory consolidation, appears to be associated with activity‐dependent synaptic plasticity in the hippocampus. Exercise induces structural neuroadaptations manifested by increased spine density in the hippocampus (Zhang et al. 2024), striatum (Ren et al. 2022), and medial prefrontal cortex (Zhang et al. 2024), while preventing VD‐associated spine degeneration. The study of synaptic plasticity thus may provide more evidence for the mechanism of exercise‐induced cognition improvement. Furthermore, the hippocampus demonstrates dual operational modes during cognition processing: either functioning as an integrated unit or isolated parts may be responsible for different functions (Lisman et al. 2018, Moser and Moser 1998). Thus, the hippocampus provides a good model for studying synaptic plasticity and cognition.

Interestingly, exercise‐induced cognitive improvements in VD models demonstrate precise parameter dependency, with therapeutic efficacy modulated by intervention duration, intensity thresholds, and modality specificity. It was reported that both 1 week and 4 weeks of exercise interventions can ameliorate the anxiety‐like behavior in VD rats by hippocampal serotonergic pathway activation (Fan et al. 2022). Comparative studies by Guo et al. (2023) demonstrate high‐intensity interval training confers superior neuroprotective efficacy versus moderate continuous training, evidenced by greater BDNF upregulation and reduction in hippocampal neuronal in VD rats (Guo et al. 2023). More importantly, different exercise types, swimming (Bashiri et al. 2020), treadmill exercise (Ohtomo et al. 2021), forced or voluntary running exercise (Rafie et al. 2023), exhibit comparable capacity to mitigate cognitive deficits in VD and other neurodegenerative diseases (Lin et al. 2015). Controversially, others’ research revealed that involuntary exercise induced by functional electrical stimulation may be as beneficial for alleviating cognitive deficits after cerebral ischemia by the BDNF‐mediated pathway (Lin et al. 2015). No direct study, however, has been performed to observe the effect of different pretreatment exercise types on hippocampal synaptic plasticity. The molecular mechanism connecting different pretreatment exercise types and cognition in the VD model is also lacking.

In an attempt to address these concerns and shortcomings, this study aimed to determine the effects of different exercise interventions, a nonpharmacological treatment that targets multiple factors and pathways. In addition, the present study was conducted to analyze the effects of long‐term exercise pretreatment on VD before pathological features were detected rather than post‐treatment. Given that VD's pathological processes may initiate decades before symptomatic onset, early exercise pretreatment intervention holds promise in delaying the emergence of VD symptoms. Therefore, this study delved into the consequences of exercise pretreatment (e.g., treadmill exercise and voluntary running) on memory retrieval and hippocampal structural synaptic plasticity and morphology in VD rats. Notably, we further compared the difference between treadmill exercise and voluntary running on the brain neuroprotective effect in the VD rat model. Our aim was to determine whether exercised‐enhanced cognition is correlated with alternations in hippocampal structural synaptic plasticity.

## 2 Materials and Methods

### 2.1 Animals

Forty‐eight male Sprague‐Dawley rats (260–290 g, aged 10 weeks) were obtained from the Experimental Animal Center of Peking University (Peking, China). They were housed under 12 h light/dark cycles (2 rats/cage) and had a sufficient amount of food and water (SPF‐degree) with standard laboratory conditions (24 ± 3°C, 55%–60% relative humidity). All the procedures were applied by the Ethics Committee of Experimental Animals of Henan Normal University (Approval Number: HNSD‐2023‐21‐1).

**FIGURE 1:**
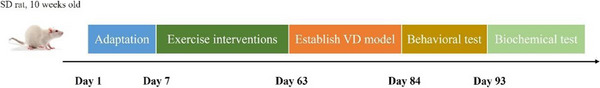
Schematic of the experimental protocol.

In this experiment, all the rats were randomly divided into the following four groups: control group (Con; n = 12), vascular dementia group (VD; n = 12), treadmill exercise + VD group (Tre‐VD; n = 12), and voluntary running exercise + VD group (Vol‐VD; n = 12). The rats in the Tre‐VD and Vol‐VD groups received treadmill exercise and voluntary exercise, respectively. After 8 weeks of exercise interventions, all the rats except the Con group received 2‐VO surgery to establish vascular dementia model. Finally, all the rats received a behavioral test and subsequent experiment. Details can be found in Figure 1. Note: during the course of the experiment, 1 rat died in the VD group. In addition, the animals did not participate in either exercise during the 21 days of VD or the 9 days of behavioral testing.

### 2.2 Exercise Interventions Protocol

Exercise interventions started after 3 days of adaption and continued for 8 weeks. All the exercise sessions were conducted from 19:00 p.m. to 21:00 p.m. per day.

In the treadmill exercise protocol, the rats in the Tre‐VD group were exercised on rodent treadmills (BHW‐PT/5 s, Anhui, China) for 60 min per day (five times per week) at a speed of 15 m/min on a treadmill equipped with moderate shock detection. An electrified grid (1 Hz, 0.46 mA) could shock rats up to 10 times if they left the moving belt while exercising. The treadmill exercise training protocol was based on procedures described elsewhere (Allen et al. 2018).

In the voluntary running protocol, the rats in the Vol‐VD group were placed in the voluntary running wheel (wheel circumference, 100 cm; ZL‐016E, Yaokun Instrument Co., Anhui, China). After 1 week of acclimatization, the rats were placed in a 100 cm diameter wheel for the subsequent 5 days a week for a total consecutive 8‐week period. Daily wheel revolutions of each rat were measured by a magnetic counter directly installed on the running wheel. The daily running distance of each rat was calculated by wheel revolutions multiplied by the wheel circumference. The volunteer running the training protocol was previously described and is also detailed in the protocol paper (Zhang et al. 2017).

### 2.3 Animal Models of VD

All the rats except for the Con group were treated with bilateral common carotid artery occlusion to establish vascular dementia rat models. Briefly, all the rats were anesthetized with 1% pentobarbital sodium. Neck skin was cleaned with 75% alcohol, and a neck midline incision using surgical instruments. The bilateral common carotid artery was carefully exposed and ligated using silk sutures to avoid damage to the vague nerve (Cho et al. 2017). During operation, the rat was placed on a heating pad to maintain body temperature at 37.0°C ∼ 37.9°C. The rat in the Con group received the same two‐step operation procedure without ligatures. Finally, 1 rat in the VD group was excluded because it died during the establishment of the VD model.

### 2.4 Assessment of Neurologic Deficit Scores

After three weeks, the “Zea‐Longa” 5‐point scale was used to evaluate postoperative neurological function and confirm whether the VD animal model had been established successfully (Yang et al. 2016). In this experiment, rats that scored at 1 to 3 points were selected and used for the next experiments. While the rats not showing behavioral deficits at the above time points were excluded from the study. It is noted that one rat died in the VD group during the BCCAO operation Table 1.

**TABLE 1:** The “Zea‐Longa” 5‐point scale.

Score	Symptom
0	No neurological deficit
1	f Ailing to fully extend left forepaw
2	Circling to the left
3	Falling to the left
4	No spontaneous walking

### 2.5 Behavioral Test

Behavioral tests, including the open field test, novel object recognition test, and passive avoidance test, were tests for cognition between 07:00 p.m. and 11:00 p.m. on day 68.

The sucrose preference test was used to investigate depression‐like status because of rats’ natural preference for sweets (Wu et al. 2020). At the training trial, all the rats were fed with two bottles of water for 2 days and a 2% sucrose solution for the subsequent 2 days. Then, the rats were deprived of food and water for 24 h. At a normal testing day, the rats were single optionally fed with two bottles of liquid, one with 2% sucrose solution and the other with water. The intake of sucrose solution and water was calculated. Sucrose preference = (sucrose intake / [water intake + sucrose intake]) × 100%.

An open field test was used to evaluate autonomic activity and anxiety‐like status. The open field test was conducted in a square open arena (100 cm × 100 cm, made of opaque plexiglass material) and monitored for 10 min. The tracking device automatically determined the frequency of immobility and the total moving distance. The researchers recorded rearing frequency, in which the rats stood on their hind leg. The tracking system calculated the time in the center zone of the rat. The following formula was used to get the center zone preference percentage: central preference % = (time in center zone / total experiment time) × 100 (Alghamdi 2022). The arena was cleaned with 75% ethanol after each rat to reduce any odor bias.

The novel object recognition test, one of the most commonly used recognition memory tests in behavioral neuroscience. The protocol consists of two days. On the first day, the rats are placed in the box (100 × 100 cm^2^, 50 cm height, made of opaque plexiglass material) for 10 min with two identical objects. When the rats were facing, sniffing, or biting the thing, it was deemed that they were exploring, and the number of the rats that explored the object was recorded. The time taken to explore each object within 10 min was also recorded. Then, the rats returned to the cage. After 24 h, the rats were re‐tested in the test box. At this stage, one of the familiar objects was replaced with a new object (only a different shape), and the rats were allowed to explore for 10 min. Memory preference was assessed using a discrimination index, which is calculated by dividing the amount of time spent with the novel object by the total amount of time spent examining either object (Zarifkar et al. 2019).

The Y‐maze was used to evaluate spatial working memory, as previously described. Briefly, each rat was given eight minutes to freely explore the maze in the arm of a symmetrical Y‐maze apparatus. The sequence and the total number of arm entrances were then manually recorded. When both hind paws were inserted within the arm, an arm entrance was verified. In this test, entering the three arms consecutively was considered one correct alternation (for instance, the pattern ABC would have three correct alternations: ABC, BCA, and ACB). The percentage of correct spatial alternation was calculated as the number of correct alternations / (total number of arm entries − 2) * 100% (Gao et al. 2022). After the test, the arms were cleaned with 75% ethanol.

The passive avoidance test was utilized to measure learning and memory function in rodents. The apparatus of the passive avoidance test is composed of a light room and a dark room with a gate between them. At the training phase, the rat was placed in the light room and allowed to freely explore between two rooms for 5 mins. During this stage, the rat received a 3 s mild foot shock with the gate closed when the rat fully entered the dark room. At the test phase (24 h after the training phase), the rat was placed again in the light room and freely explored for 5 min. During this process, without electric foot shock, the rat went from the light room to the dark room. The latency was defined as the time spent by the rat from the light room to the dark room. If the rat doesn't enter the dark room for 300 s or more, it is recorded as 300 s (Yang et al. 2022).

### 2.6 Neurotransmitter Test

Extracellular neurotransmitter level in the hippocampus was collected by a brain microdialysis combined with a high‐performance liquid chromatography (HPLC) system, including epinephrine (E) and norepinephrine (NE) as well as dopamine (DA). The microdialysis probe (CMA/12, Stockholm, Sweden) was implanted into the left hippocampus, and then continuously perfused with artificial cerebrospinal fluid (pH = 7.0) by a microinjection pump. After equilibrating for 90 min, the brain extracellular fluid was collected every 10 min into a 1.5 mL EP tube. After microdialysis experiments, HPLC was processed to quantify hippocampal neurotransmitter level. 20 µL of extracellular fluid was auto‐injected onto a chromatographic column (Waters Corporation, Milford, MA, USA). The flow rate of the mobile phase used for compound separation was 0.6 mL/min (Hu et al. 2015).

### 2.7 Immunofluorescent Staining

After being perfused with pre‐cooled PBS solution, the three whole brains were immersed in 30% sucrose solution for dehydration. Then the brain tissues were longitudinally sliced 20 µm thick on a frozen microtome (Thermo, USA) at ‐20°C. The following primary antibodies were used for immunostaining: rabbit anti‐NeuN monoclonal antibodies (ab177487, Abcam, 1:200), and incubated overnight at 4°C and then washed three times with PBS. Then they were incubated with fluorescein‐goat anti‐rabbit second antibody (ZF‐0317, Zhongshanjinqiao) for 90 min at room temperature. Images were acquired using a Nikon fluorescence microscope (Nikon model E‐600, Nikon, Kawasaki, Japan). ImageJ and Photoshop software were mainly used for immunofluorescent staining data analysis (Kim et al. 2020). Notably, to provide a single value for all rats, the fluorescence intensity of each brain slice was averaged. Additionally, the same image acquisition parameters were used for all of the photos.

### 2.8 TUNEL Staining

As previously described, the transferase‐mediated dUTP‐biotin nick end labelling (TUNEL) staining was conducted to mark the apoptotic neurons and performed following the manufacturer's instructions. Briefly, brain sections were fixed in 4% paraformaldehyde (20 min, 37°C), and washed with PBS. Then, brain sections were permeabilized with 0.5% Triton X‐100 to make the cell membrane permeable, and blocked with 5% bovine serum albumin (1 h, 37°C). After PBS washing, they were incubated overnight at 4°C with anti‐α‐actinin (1:200) in a humid chamber overnight, followed by Cy3‐conjugated secondary antibody (1:400) for 2 h at 37°C. The brain sections were incubated with TUNEL reaction mixture and then stained with DAPI. Finally, the TUNEL‐positive cells were quantified by fluorescence microscopy to assess vascular dementia‐induced apoptosis.

### 2.9 Transmission Electron Microscopy

Rat brains (6 per group) were removed following transcardial perfusion with 4% polyformaldehyde perfusion, quickly separated left hippocampal from the brain, and rinsed with normal saline. Hippocampal tissues were rapidly cut into small blocks (no more than 1 mm^3^), then the small tissue blocks were transferred into an EP tube with fresh TEM fixative and kept at 4°C until use. The tissue blocks were fixed with 2.5% (w/v) glutaraldehyde overnight, washed three times for 10 min in PBS, and then soaked with 1% osmic acid at 37°C for 3 h. After washing three times, the tissues were taken out and rinsed three times with PBS, then dehydrated with 50%, 70%, 90% alcohol, 90% alcohol + 90% acetone, 90% acetone at 4°C, and then 100% acetone at room temperature. After dehydration, the tissues were embedded in the mixture of acetone and entrapped liquid (2:1) for incubation at room temperature for 4 h, followed by acetone and entrapped liquid (1:2) for incubation at room temperature overnight, and then moved into entrapped liquid at 37°C for 4 h. After that, the tissue was incubated at 37°C for overnight and 45°C for 12 h as well as 60°C for 48 h. The coronal sections were obtained by ultra‐microtome. Synaptic ultrastructure was observed using the transmission electron microscopy (Hitachi, Tokyo, Japan) (Cheng et al. 2020).

### 2.10 Statistical Analyses

All of the study's data were reported as mean ± SEM for statistical analysis. Using the Shapiro‐Wilk test, the resulting data's normality was examined for each variable. Data analyses were statistics by SPSS24.0 and calculated by GraphPad Prism 8.0.2. A *p* < 0.05 was defined as statistical significance.

## 3 Results

### 3.1 Total Exercise Distance

**FIGURE 2:**
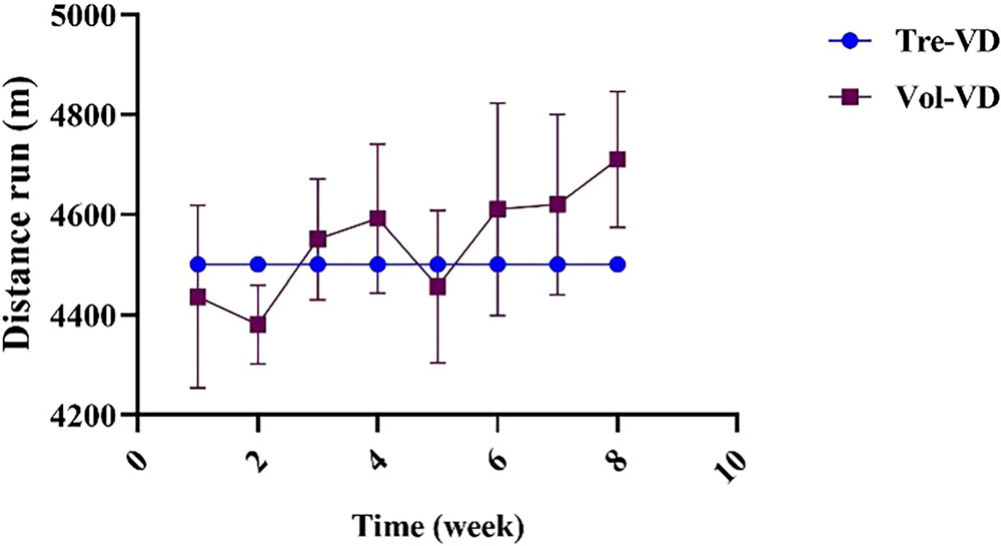
Mean hourly distance run (m) during consecutive 8 weeks exercise interventions. *N*=11‐12 rats per group.

The average running distances of the Tre‐VD and Vol‐VD groups per week are shown in Figure 2. The mean weekly distance run in the Vol‐VD group was 4544.63 ± 103.77 m, which was consistent with previous results (Greenwood et al. 2011). In addition, analyses revealed that no group differences were observed in the amount of running distance per week between the Tre‐VD and Vol‐VD groups (*p* > 0.05).

### 3.2 VD Model Evaluation

**FIGURE 3:**
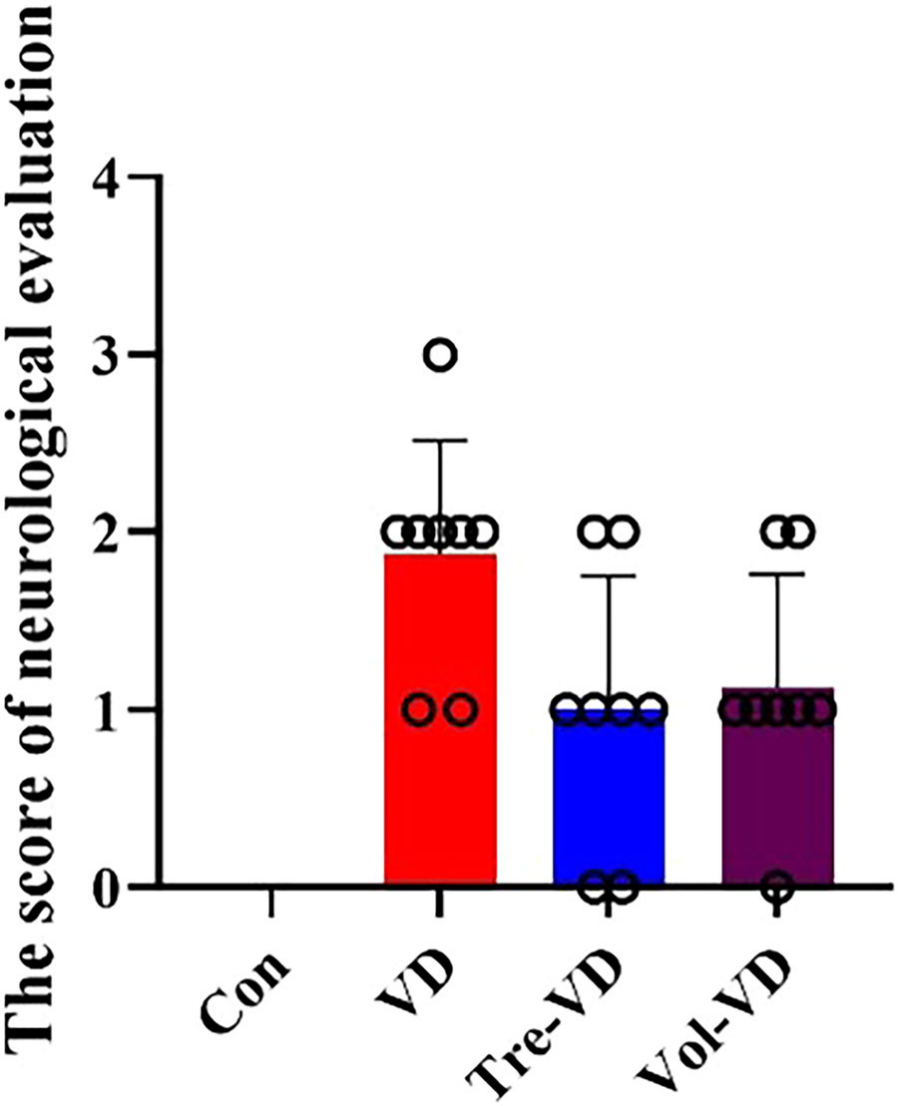
Neurological score evaluation in each group. *N* = 11–12 rats per group.

As shown in Figures 2 and 3, rats in the Con group had no neurological deficit. The VD model rats failed to fully extend the left forepaw or circled to the left or failed to the left, and the neurological function scores of the VD group significantly improved compared with the Con group (*p* < 0.05). However, treadmill exercise and voluntary running interventions improved the behavior, and neurological responses of the Tre‐VD and Vol‐VD groups significantly improved compared with the VD group (*p* < 0.05).

### 3.3 Effect of Different Exercise Interventions on Anxious‐Depressive–Like Behavior With Behavioral Test in VD Model

**FIGURE 4:**
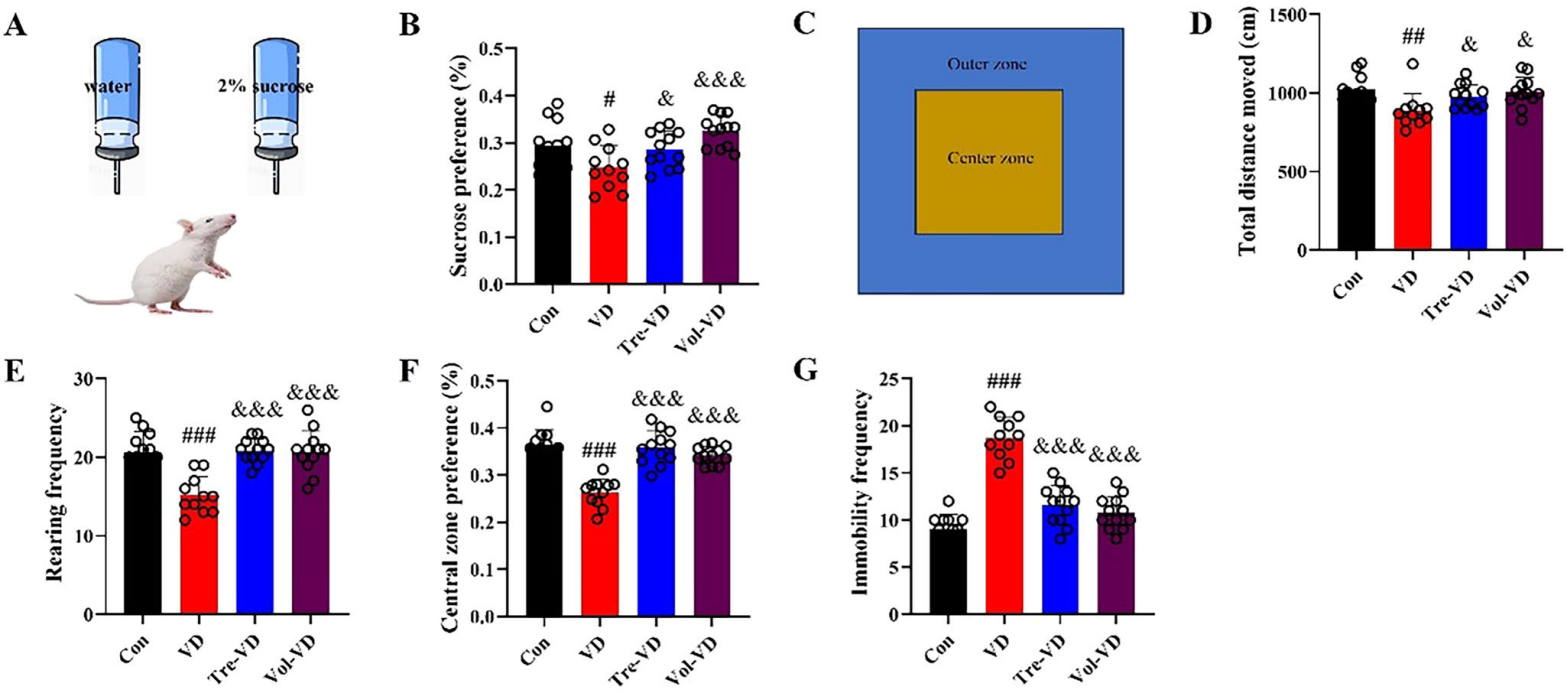
Different types of exercise training attenuated anxious‐depressive‐like behavior of VD rats. (A,B) The sucrose preference test was performed to measure depressive‐related behavior. (C–G) The open field test was performed to measure anxious‐related behavior. *N* = 11–12 rats per group.

We first investigated the role of exercise pretreatment on anxious‐depressive‐like behavior of VD rat using sucrose preference test and open field test based on the rat's natural preference. As shown in Figure 4A,B, the rats in the VD group exhibited a significantly decreased preference for sucrose as compared to the Con group (*p* < 0.05). Compared with the VD group, the sucrose preference of the Tre‐VD and Vol‐VD groups was significantly increased (*p* < 0.05), indicating that VD rats had depression‐like behaviors that were alleviated by long‐term treadmill exercise and voluntary running training.

Additionally, in the open field test (Figure 4C–G), VD rats exhibited significantly decreased total moving distance, rearing frequency, and central zone preference (*p* < 0.05), while immobility frequency was significantly increased (*p* < 0.05) compared with rats from the Con group, features which were significantly alleviated in rats undergoing treadmill exercise and voluntary running training (*p* < 0.05). Taken together, these data indicate that treadmill exercise and voluntary running training pretreatment significantly attenuate anxious‐depressive‐like behavior of VD rats.

### 3.4 Effect of Different Exercise Interventions on Cognition With Behavioral Test in VD Model

We also investigated the effect of different types of exercise training on cognitive function in VD rats using a battery of behavioral tests.

First, we examined the recognition memory of rats through the novel object recognition test. As expected, VD rats showed a lower preference for the novel object and total exploration time (*p* < 0.05); however, the rats in the Tre‐VD and Vol‐VD groups showed a better preference for the novel object and total exploration time (*p* < 0.05), which indicated that treadmill exercise and voluntary running training pretreatment effectively attenuated recognition memory impairment in VD rats.

Furthermore, the Y‐maze test further confirmed the spatial working memory in VD animals. Spontaneous alternation and the frequency of arm entries were significantly decreased in the VD group compared to that in the Con group (*p* < 0.05); in contrast, compared with the VD group, the Tre‐VD and Vol‐VD groups showed significant increase in spontaneous alternation and the frequency of arm entries (*p* < 0.05), suggesting VD animals had spatial working memory impairments that were alleviated by treadmill exercise and voluntary running training.

**FIGURE 5:**
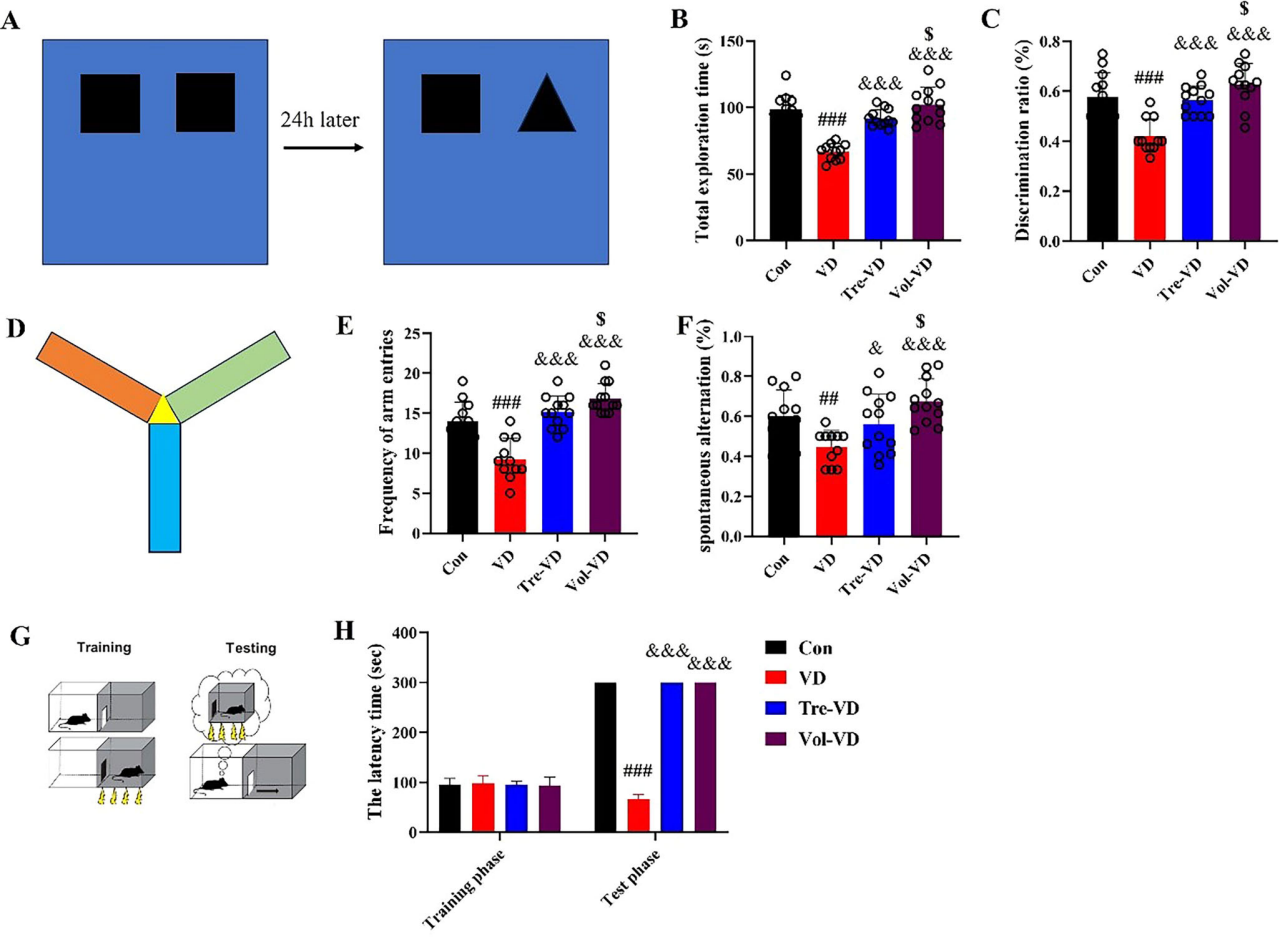
Different types of exercise training attenuated cognitive function impairment in VD rats. (A–C) The novel object recognition test was used to test recognition memory; (D–F) the Y‐maze was conducted to test spatial working memory; (G–H) the passive avoidance test was performed to measure learning and memory function. *N*=11‐12 rats per group.

**FIGURE 6:**
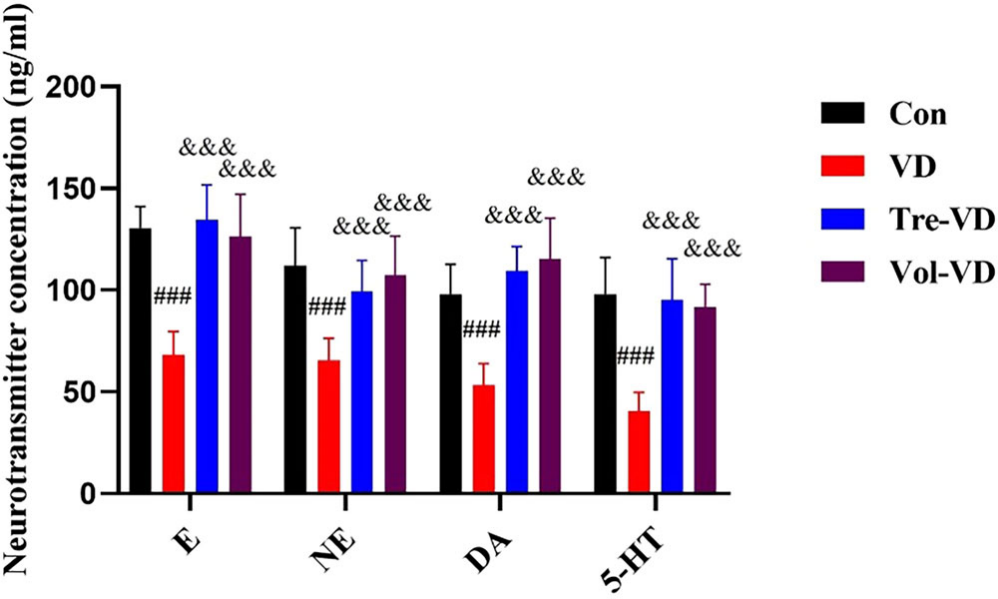
Expression of neurotransmitters on the hippocampus region in each group. The concentration of (A) E, (B) NE, (C) DA, and (D) 5‐HT. *N* = 11–12 rats per group.

Moreover, the passive avoidance test was performed to measure learning and memory function. As shown in Figure 5A–C, at the training phase, there were no significant differences among each group in the latency of entrance to the dark room (*p* < 0.05), revealing that they were at the same cognition level before the electrical shock. Figure 6 Furthermore, in the test phase, the latency time in the VD group exhibited a remarkable decrease as compared with Con rats (*p* < 0.05), which suggests that VD rats forget the electric stimulus when they go from the light room to the dark room, indicating a poor learning and memory function of VD rats. Conversely, compared with the VD group, the Tre‐VD and Vol‐VD groups exhibited remarkable increased latency time (*p* < 0.05), which suggests that treadmill exercise and voluntary running training pretreatment significantly improved learning and memory in VD rats.

Taken together, our results show that long‐term treadmill exercise and voluntary running training pretreatment are capable of attenuating cognitive impairment in VD rats.

Together, these results reveal that TLB effectively rescues cognitive deficits of AD animal models, and HMGB1 might be a pivotal factor involved in the neuroprotective effects of TLB on AD.

### 3.5 Effect of Different Exercise on Neurotransmitter Level in VD Model

To investigate whether exercise‐induced reduction in cognitive function was associated with hippocampal neurotransmitter level, we quantified neurotransmitter level parameters of the hippocampus that are critical for the transmission of information related to cognitive function. Compared to the Con group, model rats (VD group) had significantly decreased E NE, DA, and 5‐HT (*p* < 0.05). Conversely, compared to the VD group, those two exercise treatment groups had increased contents of those 4 neurotransmitters (*p* < 0.05), revealing that the rats in the Tre‐VD and Vol‐VD groups had elevated neurotransmitter levels after exercise pretreatment. These data supported that treadmill exercise and voluntary running pretreatment rescued hippocampal neurotransmitter levels in VD rats.

### 3.6 Effect of Different Exercise on Brain Neuron in VD Model

**FIGURE 7:**
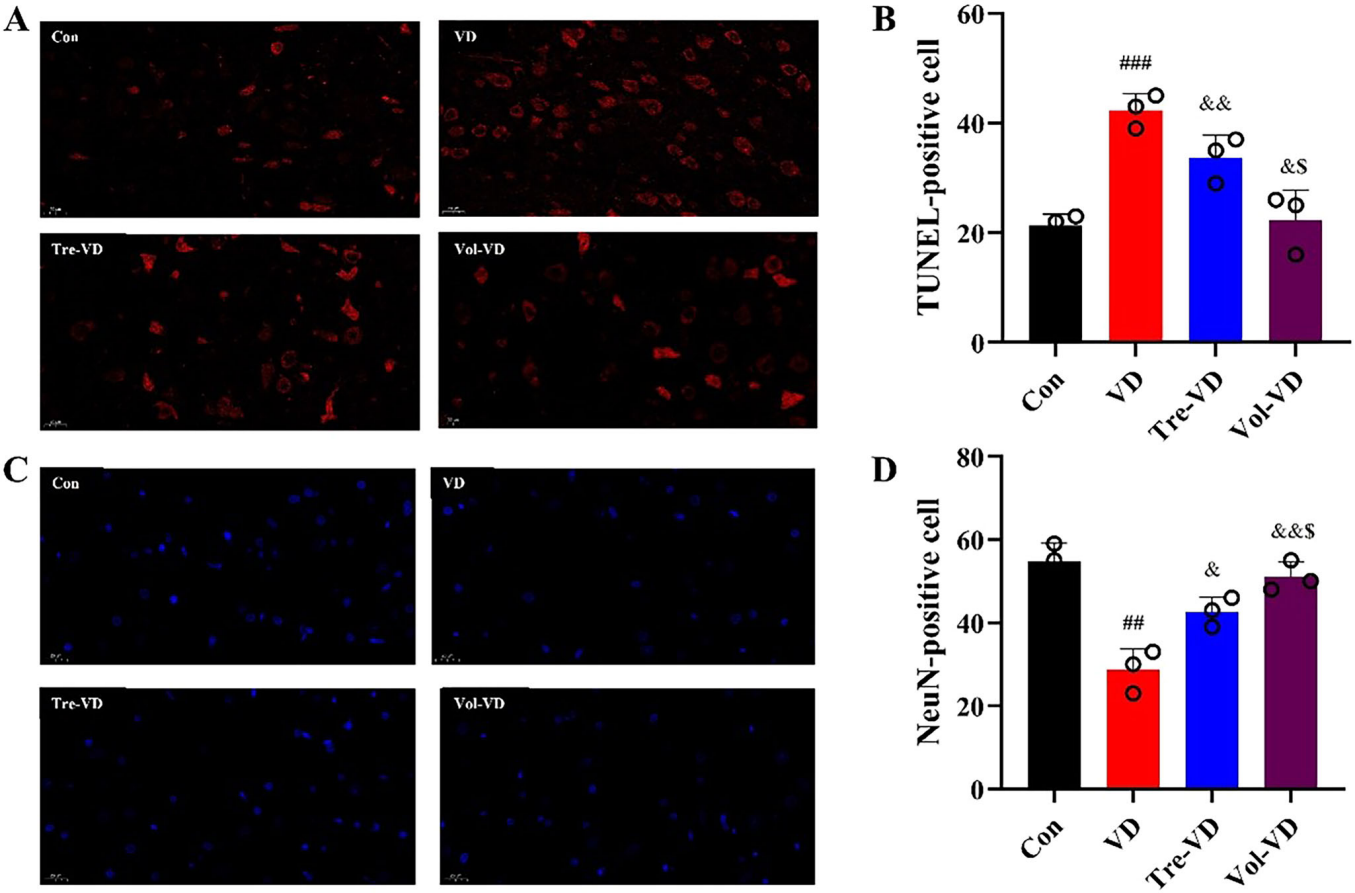
Exercise pretreatment alleviates neuronal damage in VD rats. (A) Representative TUNEL staining images, (B) the number of neuronal apoptosis in the hippocampus, (C) representative NeuN immunofluorescent staining images, and (D) the number of NeuN‐positive neurons in the hippocampus. Scale bar = 20 µm. *N* = 3 rats per group.

We next further examine the effects of exercise pretreatment on neuronal neuroprotection and apoptosis in VD rats. TUNEL staining was used to analyze whether exercise pretreatment could ameliorate neuronal apoptosis. As presented in Figure 7B, VD‐induced significantly increased TUNEL‐positive neurons in the hippocampus compared to Con rats (*p* < 0.05). However, exercise pretreatment effectively alleviated VD‐induced neuronal apoptosis, as evidenced by the fact that the number of TUNEL‐positive neurons was significantly decreased in Tre‐VD and Vol‐VD rats as compared with VD animals (*p* < 0.05), and the number of TUNEL‐positive neurons in the Vol‐VD rats was significantly decreased as compared with that of the Tre‐VD rats (*p* < 0.05). These findings reveal that treadmill exercise and voluntary running pretreatment protect against neuronal apoptosis in hippocampal neurons, and this protective effect of voluntary running is better than treadmill exercise.

To further confirm these results, NeuN immunofluorescent staining was performed to analyze whether exercise pretreatment could protect against neuronal apoptosis. Figure 7D demonstrated that VD rats exhibited significantly decreased NeuN‐positive neurons compared to Con rats (*p* < 0.05). In contrast, the Tre‐VD and Vol‐VD rats showed significantly more NeuN‐positive neurons than those in the VD group (*p* < 0.05), and further analysis revealed that the increased phenomenon of hippocampal NeuN‐positive neuron numbers in the Vol‐VD group is better than in the Tre‐VD group (*p* < 0.05). These findings indicate that treadmill exercise and voluntary running pretreatment protect hippocampal neurons, and the positive effect of voluntary running on hippocampal neurons is better than treadmill exercise.

### 3.7 Effect of Different Exercise on Synaptic Ultrastructure in VD Model

**FIGURE 8:**
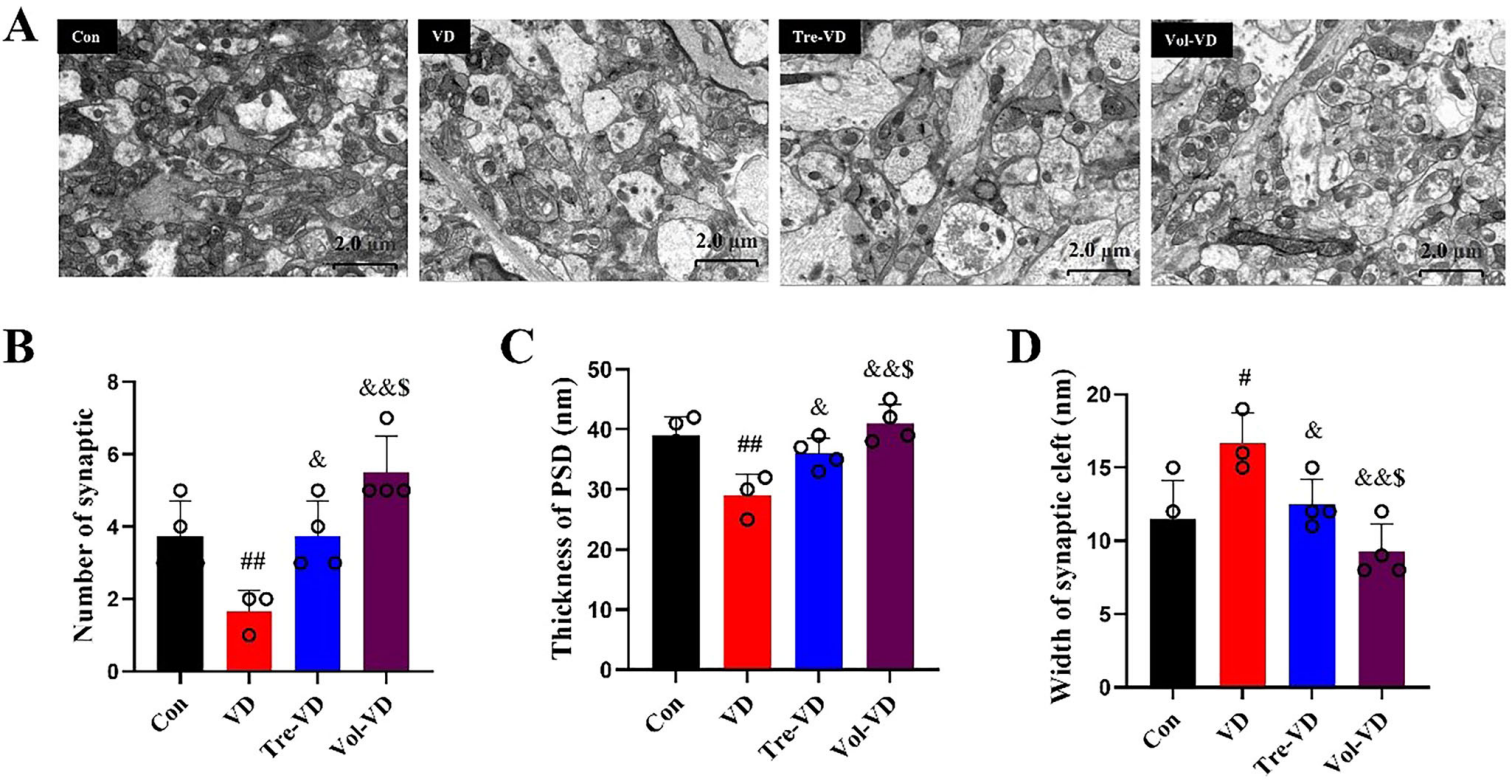
Exercise pretreatment ameliorated synaptic pathology of the hippocampus in VD rats. (A) Representative transmission electron microscope photos in each group, (B) the number of synaptic, (C) the thickness of the postsynaptic density, and (D) the width of the synaptic cleft. Scale bar = 2.0 µm. *N* 3–4 rats per group.

To evaluate synaptic transmission efficacy in the rat hippocampus, we performed quantitative ultrastructural analysis using TEM, including the number of synaptic, the thickness of the postsynaptic density, and the width of the synaptic cleft (Figure 5A ). We can learn from Figure 5B that there was a significant decrease in hippocampal synapse numbers in VD rats compared to the Con group (*p* < 0.05), which was reversed by 8‐week exercise pretreatment. Specifically speaking, compared with the VD group, the synapse numbers of the hippocampus in the Tre‐VD and Vol‐VD groups were significantly increased (*p* < 0.05), and further analysis revealed that the increased phenomenon of synapse numbers in the Vol‐VD group is better than in the Tre‐VD group Figures 6, 7 and 8.

Regarding the thickness of the postsynaptic density, the analysis of Figure 8C revealed that VD rats exhibited thinner PSDs compared to those of the Con group (*p* < 0.05); they thickened significantly in the Tre‐VD and Vol‐VD groups after exercise (*p* < 0.05), and the thickening phenomenon of postsynaptic density in the Vol‐VD group was greater than in the Tre‐VD group (*p* < 0.05).

The width of the synaptic cleft was lastly detected. Figure 8D revealed that the VD rat had wider clefts than the Con rat (*p* < 0.05), which were significantly narrowed by exercise pretreatment in the Tre‐VD and Vol‐VD groups (*p* < 0.05), and the narrowing phenomenon of synaptic cleft in the Vol‐VD group was greater than in the Tre‐VD group (*p* < 0.05). Such evidence collectively supported that treadmill exercise and voluntary running can all improve synaptic transmission efficiency in VD rats, and the positive effect of voluntary running is better than treadmill exercise.

## 4 Discussion

Our study demonstrates that exercise pretreatment alleviates cognitive impairment in VD rat models and provides neuroprotection against neurodegeneration. Further mechanistic studies revealed that the neuroprotection effects of exercise pretreatment on VD might be due, in part, to exercise pretreatment, all of which can improve hippocampal structural synaptic plasticity in VD rats. Specifically speaking, long‐term exercise pretreatment alleviates memory impairment and anxious‐depressive‐like behavior in VD rat models by protecting against hippocampal neurotransmitter decrease, neuronal apoptosis, rescuing synapse numbers, postsynaptic density, and synaptic cleft width in VD rats. Furthermore, we also compared the difference between treadmill running and voluntary running on the brain neuroprotective effect in the VD rat model. The current study, to our knowledge, provides the first piece of in vivo evidence showing that the positive brain effect of voluntary running on hippocampal structural synaptic plasticity is better than treadmill exercise. Our studies provided additional evidence that long‐term treadmill exercise and voluntary running pretreatment, especially voluntary running, are potential approaches to delay the progression of VD.

It is necessary to emphasize that our experiment enrolled healthy adult male SD rats as subjects for the following reasons: (1) to control for interference from sex hormones. Female rats experience periodic fluctuations in hormone levels (estrogen, progesterone) due to the estrous cycle, which may affect experimental outcomes (e.g., metabolism, behavior, or neuroplasticity). In contrast, male hormones (testosterone) remain relatively stable, making it easier to control variables. (2) To avoid pregnancy‐related complications. Accidental pregnancy in female animals could disrupt experiments and raise ethical concerns (e.g., fetal rights). (3) To reduce physiological variability. Adult male rats exhibit more consistent physiological metrics (e.g., body weight, organ function), minimizing intra‐group variation and improving statistical power. Thus, we adopted healthy adult male SD rats as experimental subjects in our study.

Exercise, a ubiquitous habit, triggers essential neuroprotective reactions for promoting cognitive performance. In vivo studies utilize various methods to assess cognitive functions, such as the Morris water maze for evaluating learning and memory (Ozkan et al. 2022), the Y‐maze for spatial working memory (Gu et al. 2017), and the novel object recognition task for recognition memory (Grayson et al. 2015). Animal data confirmed the positive effect of exercise on learning and memory, spatial working memory, and recognition memory, as well as pattern separation‐dependent memory on VD and other disease models (Choi et al. 2018, Biose et al. 2024, Liang et al. 2023). Consistent with previous studies, our results also demonstrated that VD rats exhibit worse cognitive function, including learning and memory, recognition memory, and spatial working memory in behavior tests. The Morris water maze task and radial arm maze supplementary revealed impaired spatial learning and memory performance in the VD model (Bayat et al. 2023, Sun et al. 2023).

Previous findings also revealed that VD rats exhibited a smaller sucrose preference coefficient in the sucrose preference test (Hu et al. 2021) and reduced movement ability (Fan et al. 2024), indicating that VD rats were likely depressed and anxious as well as had a movement disorder. Our observations concurred with prior reports, demonstrating cognitive impairment and anxious‐depressive‐like behavior in the VD model. Remarkably, 8‐week exercise interventions ameliorated cognition impairments in the VD rat.

Notably, the positive effects of various types of physical exercise on cognition and emotion have been acknowledged in VD and other dementia types. Treadmill and wheel running exercises, as classic models of forced and voluntary exercise, respectively, were employed in the present study. More specifically, treadmill exercise or voluntary running pre‐treatment protocols were implemented at 11 weeks of age, preceding the typical onset timeline of VD symptoms. We demonstrated that treadmill exercise and voluntary running pretreatment can all ameliorate cognitive impairment and anxious‐depressive‐like behavior of VD rats. This, coupled with findings from exercise in other VD models (Lee et al. 2019, Dao et al. 2024), indicates its potential as an early intervention to delay VD progression. Unfortunately, our findings did not demonstrate behavioral differences between forced treadmill and voluntary running exercises. Therefore, whether treadmill and voluntary running exercises differ in other aspects for VD rats requires further investigation.

Cerebral neuronal encoding may underlie the processes of memory formation and emotional modulation (Sun et al. 2020). Histological analysis of VD sections using NeuN (Chen et al. 2021) and TUNEL staining (Ningning et al. 2024) demonstrated reduced NeuN‐positive neuronal populations and elevated TUNEL‐positive neurons in treatment groups relative to controls. In line with previous studies, our data demonstrated that VD rats developed marked neurodegenerative pathology following VD administration, characterized by elevated TUNEL‐positive neuronal apoptosis and reduced NeuN‐positive neuronal density. Conversely, both exercise modalities effectively mitigated these pathological alterations. The mechanism underlying exercise‐inhibited neuronal damage can be illustrated from three aspects. First, the linkage of exercise ameliorating neuronal damage by regulating neurotrophic factors (eg., BDNF, NT‐3) expression has also been investigated. Ketone body beta‐hydroxybutyrate, the metabolite produced after exercise or exogenous introduction of BDNF (Choi et al. 2018) interventions, has been shown to enhance BDNF expression (Sleiman et al. 2016). Second, exercise has been shown to inhibit inflammation level. A series of human studies revealed inhibiting effects of physical exercise on inflammation level. In major dementia patients, aerobic exercise has been shown to ameliorate inflammatory response and improve symptoms (De Miguel et al. 2021, Zhao 2024). Third, the linkage between exercise and mitochondria has been systematically elucidated, with exercise‐improved mitophagy and mitochondrial function (Liang et al. 2021), and suggests that mitochondria are targets for enhancing neurogenesis‐dependent hippocampal plasticity. Interestingly, our histological analysis further revealed that voluntary running exercise demonstrates superior neuroprotective efficacy compared to treadmill exercise in VD rats. That phenomenon can be explained by the fact that voluntary exercise is the most effective intervention compared to treadmill exercise and involuntary exercise regimens, and rats that exercised in the voluntary wheel also showed less corticosterone stress response than those that did treadmill exercise and involuntary exercise (Hare 2014). These results also suggested that the forced exercise group was the least preferred intervention, with high stress and less motor recovery.

Hippocampal structural plasticity is dynamically orchestrated by synaptic ultrastructural remodeling, entailing quantitative modulation of synaptic density, nanoscale reconfiguration of postsynaptic density (PSD) thickness, and precise regulation of synaptic cleft dimensions (Südhof 2021). Specifically, (1) synapses serve as the fundamental and indispensable structural platform for inter‐neuronal information transmission (Mansilla et al. 2018). Our results revealed significant alterations in the number of the synapses of VD rats, marked by a reduction in the number of hippocampal synapses, while treadmill exercise improved the synaptic number in VD rats. This finding concurred with the previous results (Zhang et al. 2024); (2) the thickness of the PSD represent postsynaptic dynamics (Shen et al. 2023). We revealed that the thickness of the PSD was exhibited thinner in the VD rats than that in the control group, while it thickness significantly increased in the exercise interventions group, suggesting that exercise exerts a protective effect on PSD thickness in VD rats; (3) the synaptic cleft serves as the essential conduit for neurotransmitter transfer signaling, functioning as a pivotal morphological determinant in inter‐neuronal communication (Tao‐Cheng et al. 2023). Our results revealed significant alterations in the hippocampal synaptic architecture of VD rats, marked by an expansion of the synaptic cleft width, alongside a reduction in the neurotransmitter concentrations. Conversely, treadmill exercise induced beneficial structural adaptations in these rats, characterized by enhancements in the hippocampal neurotransmitter concentration and a narrowing of the synaptic cleft, aligning with prior observations by Zhang et al. (2020) and Yu et al. (2024). In particular, the TEM results showed that voluntary running exerts a better protective effect on hippocampal synaptic plasticity than treadmill exercise. Ultrastructural analysis via TEM revealed that voluntary running confers enhanced neuroprotective efficacy in hippocampal synaptic ultrastructural preservation compared to treadmill exercise. Voluntary running is categorized as a non‐stressogenic exercise modality that circumvents hypothalamic‐pituitary‐adrenal (HPA) axis activation, whereas controlled treadmill exercise demonstrably provokes significant corticosterone surges indicative of neuroendocrine stress‐axis engagement (Ke et al. 2011). Arida's experimental data concur with our findings demonstrating that voluntary running induces more pronounced neuroplastic alterations, particularly synaptic remodeling within the hippocampal dentate gyrus's granule cell layer (Arida et al. 2004).

Taken together, our study provides clear evidence that treadmill exercise and voluntary running are more effective in counteracting anxious‐depression‐like status and in preventing early synaptic deficits, ameliorating motor and cognitive disturbances in VD animals. Moreover, the study underscores voluntary running regimens as a more effective countermeasure for mitigating neurological damage caused by VD. A number of important limitations of the present study need to be considered. First, we only examined the synaptic ultrastructure in whole hippocampal tissues; therefore, we cannot elucidate these changes with dendritic modifications in specific hippocampal subregions. Consequently, our study was restricted to examining two specific exercise protocols, which potentially constrains the capacity to disentangle differential neuroprotective mechanisms among exercise regimens in VD rodent models. These methodological limitations highlight the need for systematically designed investigations incorporating diverse exercise paradigms to establish evidence‐based conclusions regarding exercise‐type‐specific therapeutic efficacy in VD pathophysiology. Furthermore, current research has primarily delineated the neuroprotective effects of exercise on cognition through hippocampal synaptic plasticity modulation. Notably, accumulating evidence implicates epigenetic regulation as a critical mediator in exercise‐driven synaptic remodeling and cognitive enhancement. Therefore, further investigations are imperative to delineate the epigenetic pathways through which exercise sustains cognitive preservation and functional optimization. Lastly, we exclusively use SD rats in our experiments. However, findings from rodent studies may not strongly translate to humans. Therefore, further research should explore human evidence regarding how exercise protects against cognitive impairment in VD patients.

## 5 Conclusions

These results demonstrate that voluntary exercise can improve both cognition and anxious‐depressive‐like behavior status in the VD cases about as well as treadmill exercise. This may provide an effective means to treat VD‐induced cognitive impairment in certain cases. The results also show that compared with forced treadmill exercise, voluntary exercise can increase the content of neurons and synaptic PSD thickness, and decrease synaptic cleft in the hippocampus, protect neurons against degeneration, and enhance synaptic plasticity.

## Author Contributions

**Yujiao Li**: conceptualization, investigation, formal analysis, writing – original draft. **Yuxuan Li**: data curation. **Linlin Zhang**: methodology. **Kunxia Su**: resources, writing – review and editing, project administration, funding acquisition.

## Peer Review

The peer review history for this article is available at https://publons.com/publon/10.1002/brb3.70833.

## Data Availability

The data that support the findings of this study are available on request from the corresponding author.
